# RefSelect: a reference sequence selection algorithm for planted (*l*, *d*) motif search

**DOI:** 10.1186/s12859-016-1130-6

**Published:** 2016-07-19

**Authors:** Qiang Yu, Hongwei Huo, Ruixing Zhao, Dazheng Feng, Jeffrey Scott Vitter, Jun Huan

**Affiliations:** 1 School of Computer Science and Technology, Xidian University, Xi’an, 710071 China; 2 School of Electronic Engineering, Xidian University, Xi’an, 710071 China; 3 Department of Computer and Information Science, The University of Mississippi, Oxford, MS 38677-1848 USA; 4 Department of Electrical Engineering and Computer Science, the University of Kansas, Lawrence, KS 66045 USA

**Keywords:** Planted (*l*, *d*) motif search, Pattern-driven, Reference sequences

## Abstract

**Background:**

The planted (*l*, *d*) motif search (PMS) is an important yet challenging problem in computational biology. Pattern-driven PMS algorithms usually use *k* out of *t* input sequences as reference sequences to generate candidate motifs, and they can find all the (*l*, *d*) motifs in the input sequences. However, most of them simply take the first *k* sequences in the input as reference sequences without elaborate selection processes, and thus they may exhibit sharp fluctuations in running time, especially for large alphabets.

**Results:**

In this paper, we build the reference sequence selection problem and propose a method named RefSelect to quickly solve it by evaluating the number of candidate motifs for the reference sequences. RefSelect can bring a practical time improvement of the state-of-the-art pattern-driven PMS algorithms. Experimental results show that RefSelect (1) makes the tested algorithms solve the PMS problem steadily in an efficient way, (2) particularly, makes them achieve a speedup of up to about 100× on the protein data, and (3) is also suitable for large data sets which contain hundreds or more sequences.

**Conclusions:**

The proposed algorithm RefSelect can be used to solve the problem that many pattern-driven PMS algorithms present execution time instability. RefSelect requires a small amount of storage space and is capable of selecting reference sequences efficiently and effectively. Also, the parallel version of RefSelect is provided for handling large data sets.

## Background

Motif discovery, as a main means to locate conserved fragments in biological sequences, is a fundamental problem in computational biology. The conserved fragments usually have special biological significance. For example, transcription factor binding sites in DNA sequences [1, 2] play a key role in gene expression regulation and they usually range from 5 to 25 base pairs; short protein sequence signatures [3, 4], which usually range from 10 to 36 residues, can be used in identifying potential interaction sites of proteins.

The planted (*l*, *d*) motif search (PMS) [5] is a famous formulation for motif discovery: given a data set *D* = {*s*_1_, *s*_2_, …, *s*_*t*_} with *t n*-length sequences over an alphabet Σ, *q* satisfying 0 < *q* ≤ *t*, and *l* and *d* satisfying 0 ≤ *d* < *l* < *n*, the goal is to find one or more *l*-length strings *m* such that *m* occurs in at least *q* sequences in *D* with up to *d* mismatches. The string *m* is called a (*l*, *d*) motif, and each occurrence of *m* is called a motif instance. Finding all (*l*, *d*) motifs present in the input sequences is NP-complete [6].

In the PMS problem, the value of *q* implies the corresponding sequence model of motif discovery (i.e., the distribution of motif occurrences in the input sequences). The usual sequence models include OOPS, ZOOPS and TCM [7], representing that each input sequence contains one occurrence, zero or one occurrence and zero or more occurrences, respectively. When *q* = *t* and 0 < *q* < *t*, the PMS problem corresponds to the OOPS and ZOOPS or TCM sequence models, respectively.

There have been numerous motif discovery algorithms [8, 9]. They are either approximate or exact, based on whether the algorithm can always find all motifs or the optimum motif. The approximate algorithms usually adopt probability analysis and statistical methods. For instance, the two classical algorithms MEME [10] and Gibbs Sampling [11] identify motifs by using expectation maximization and Gibbs sampling techniques, respectively. In general, these approximate algorithms can solve the problem in a short time but cannot guarantee global optimum.

In this paper, we mainly focus on exact motif discovery algorithms, which can find all (*l*, *d*) motifs by traversing the whole search space. The main indicator to assess exact algorithms is time performance, and researchers usually compare exact algorithms on the challenging PMS problem instances, for which the expected number of random (*l*, *d*) motifs present in the sequences is more than 1 [12]. For the exact algorithms proposed in earlier years, such as WINNOWER [5], DPCFG [13] and RecMotif [14], their search space is composed of (*n* – *l* + 1)^*t*^ possible alignments of motif instances. In recent years, the exact algorithms verify all the *l*-length patterns in the *O*(|*∑*|^*l*^) search space, and then output the patterns with motif property; we call them the pattern-driven PMS algorithms [15–24].

The pattern-driven PMS algorithms have better time performance than other exact algorithms so far in identifying both short motifs and long motifs with weak signal. Their basic idea is to generate candidate motifs by using several reference sequences in the input, and then verify each candidate motif one by one. Specifically, they generate candidate motifs by using all possible *h*-tuple *T* = (*x*_1_, *x*_2_… *x*_*h*_) composed of *h l*-length strings coming from *h* distinct reference sequences. In existing pattern-driven PMS algorithms, *h* is 1 for PMSP [15] and PMSPrune [16]; *h* is 2 for StemFinder [17], PairMotif [18], qPMS7 [19] and TravStrR [20]; *h* is 3 for iTriplet [21] and PMS5 [22]; for PMS8 [23] and qPMS9 [24], *h* is greater than or equal to 3 and self-adaptive in dealing with different PMS problem instances. Moreover, these algorithms use *k* = *t* – *q* + *h* reference sequences to generate candidate motifs, ensuring that there exists at least one *h*-tuple *T* so that each *l*-length string in *T* is a motif instance.

Although pattern-driven PMS algorithms outperform other exact algorithms, most of them use the first *k* sequences in the input as reference sequences, without considering the effect of different reference sequences on time performance. In this study we found that given a data set, different reference sequences may lead to quite different number of candidate motifs, especially for large alphabets. So, in dealing with different inputs with the same scale, the pattern-driven PMS algorithms may exhibit sharp fluctuations in running time. For instance, we randomly generate multiple groups of data sets with |∑| = 20, *t* = 20, *q* = 20 and *n* = 600 following the method described in the results and discussion section. When solving the (19, 9) problem instance, qPMS7 sometimes consumes 6.1 minutes, but sometimes over 48 hours. Some other pattern-driven PMS algorithms like TravStrR and PMS8, suffer from the same problem (see Supplement 1 for more examples).

To solve this problem, we propose a method named RefSelect to quickly select reference sequences that generate a small number of candidate motifs. RefSelect can bring a practical time improvement of the state-of-the-art pattern-driven PMS algorithms, without doing any modifications to them.

## Methods

### Problem description and notations

#### Reference sequence selection problem

Given a data set *D =* {*s*_1_, *s*_2_, …, *s*_*t*_} over an alphabet Σ that contains *t* sequences of length *n*, the (*l*, *d*) problem instance (0 ≤ *d* < *l* < *n*) and the number of reference sequences *k* (1 < *k* < *t*) required by the pattern-driven PMS algorithms, the task is to select *k* reference sequences from *D* to form the reference sequence set *D*', such that when using *D*' the pattern-driven PMS algorithms can efficiently solve the (*l*, *d*) problem instance without sharp fluctuations in running time.

In the reference sequence selection problem the value of provided *k* should be greater than 1. If *k* is 1, it means that the candidate motifs will be generated from multiple single *l*-mers; in this case, no matter how we select a reference sequence, the number of generated candidate motifs is fixed. In fact, *k* is greater than 1 for all the efficient and recently proposed pattern-driven PMS algorithms.

We evaluate a reference sequence selection algorithm from two perspectives. One is the time performance. The time cost of the reference sequence selection algorithm should be as small as possible because it is a preprocessing for pattern-driven PMS algorithms. It will be meaningless if it costs too much time to select reference sequences. The other is validity, namely whether the reference sequence selection algorithm brings a good speedup for pattern-driven PMS algorithms. The speedup is the ratio of *T*_1_ to *T*_*rs*_ + *T*_2_, where *T*_1_ is the running time of the pattern-driven PMS algorithms on the input sequences of original order, *T*_*rs*_ is the running time of the reference sequence selection algorithm, and *T*_2_ is the running time of the pattern-driven PMS algorithms on the input sequences of new order generated by the reference sequence selection algorithm.

Table 1 summarizes the notations used in this paper. Notice that, a sequence specially refers to an *n-*length string in a data set, and an *l*-mer refers to a short string of length *l* (*l* < *n*).

**Table 1:** Notations used in this paper

Notation	Explanation
*|x|*	The length of a string, the size of a set, or the number of elements in a matrix.
*D*, *D*'	*D* is the set of input sequences. *D*' is the set of reference sequences. *D* = {*s*_1_, *s*_2_, …, *s*_*t*_} and *D*' = {*s*_*r*1_, *s*_*r*2_, …, *s*_*rk*_}, satisfying *D*' ⊂ *D*.
*t*	The number of sequences in the input sequence set *D*, namely |*D*| = *t*.
*k*	The number of required reference sequences, namely |*D*'| = *k*.
*n*	The length of each input sequence.
*x* ∈_*l*_ *s*	The string *x* is an *l*-length substring of the sequence *s*. In other words, *x* is an *l*-mer in the sequence *s*.
*s*[*i*]	The *i*th character in the string *s*.
*s*[*i…j*]	A substring of the string *s* starting from the *i*th position to the *j*th position.
*d*_*H*_(*x*, *x*')	The Hamming distance between two strings *x* and *x*' of the same length.
*M*_*d*_(*x*, *x*')	The common candidate motifs of two *l*-mers *x* and *x*'. *M*_*d*_(*x*, *x*') = {*y*: |*y*| = |*x*| = |*x*'|, *d*_*H*_(*y*, *x*) ≤ *d*, *d*_*H*_(*y*, *x*') ≤ *d*}.
*N*_*r*_(*D*')	The number of candidate motifs generated from the reference sequences set *D*', calculated by (1).
*N*_*r*_(*s*_*i*_ *, s*_*j*_)	The number of candidate motifs generated from two sequences *s*_*i*_ and *s*_*j*_, calculated by (2).
min(*i*, *j*)	The minimum value between two integers *i* and *j*. min(*i*, *j*) = *i* if *i* ≤ *j*, *j* otherwise.
*sim*(*s*_*i*_ *, s*_*j*_)	The similarity of two sequences *s*_*i*_ and *s*_*j*_.

### Overview of RefSelect

We introduce why and how to select reference sequences for the pattern-driven PMS algorithms. Let us consider the following two observations, which indicate how the Hamming distance between pairs of *l*-mers affects the number of candidate motifs. Examples and detailed discussion for the two observations are given in the results and discussion section.

Observation 1. For two *l*-mers *x* and *x*', the smaller their Hamming distance *d*_*H*_(*x*, *x*'), the larger the number of their common candidate motifs |*M*_*d*_(*x*, *x*')|.

Observation 2. For a tuple *T* of *h l*-mers, when it contains pairs of *l*-mers with a relatively small Hamming distance, it generates a relatively large number of candidate motifs.

Based on the two observations, different reference sequences may lead to different number of candidate motifs. The pattern-driven PMS algorithms utilize all tuples of *h l*-mers in *k* (0 < *h* ≤ *k*) reference sequences to generate candidate motifs. Once there are relatively more pairs of *l*-mers with small Hamming distance in these *h-*tuples, more candidate motifs will be generated.

**Fig. 1:**
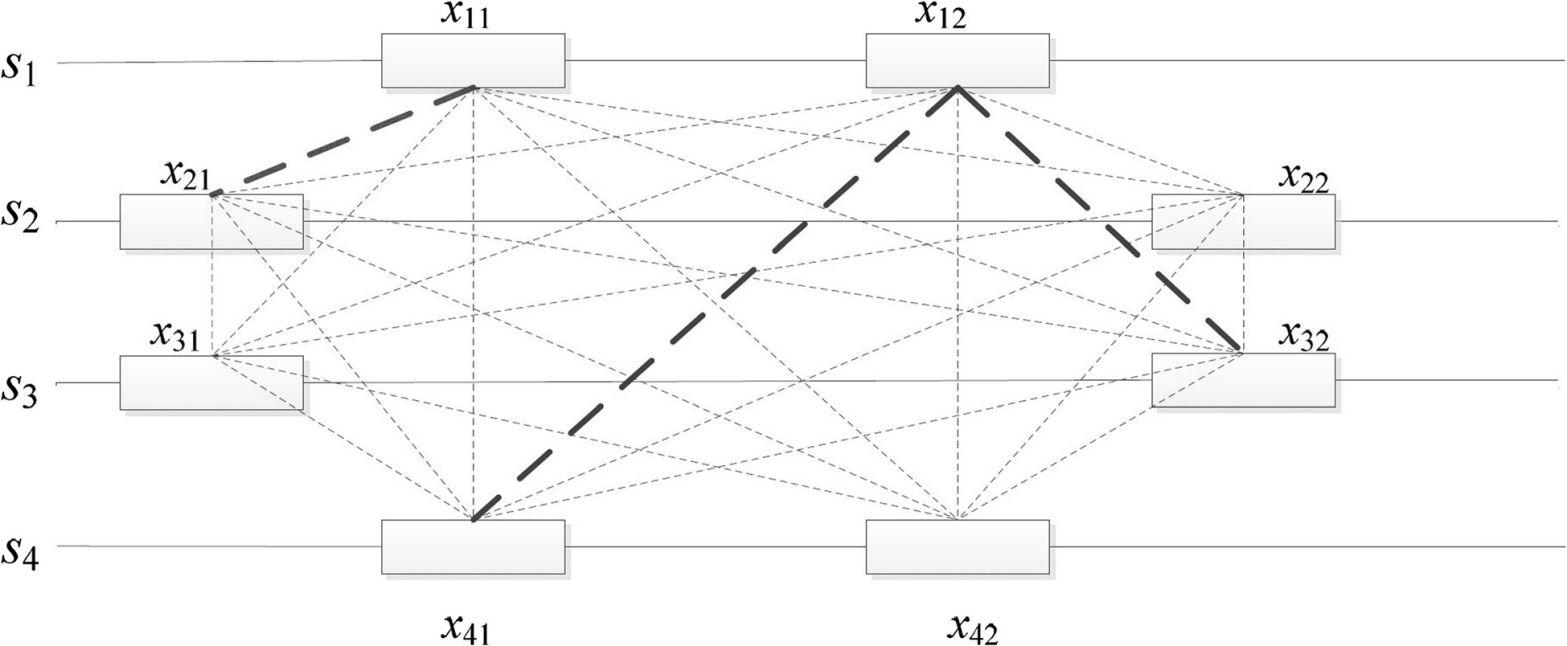
An example of selecting reference sequences. In this example, we select three reference sequences from four input sequences. Each sequence contains two *l*-mers. A thick line indicates that more candidate motifs are generated by the associated two *l*-mers

Since the time performance of pattern-driven PMS algorithms mainly depends on the number of generated candidate motifs, we should select the reference sequence set generating a small number of candidate motifs. For example, as shown in Fig. 1, assume the input sequence set *D* is {*s*_1_, *s*_2_, *s*_3_, *s*_4_} where each sequence has two *l*-mers and we select *k* = 3 reference sequences from *D*. In the figure, the thicker the dotted line, the more candidate motifs are generated by the associated two *l*-mers. Obviously, {*s*_2_, *s*_3_, *s*_4_} is the optimal reference sequence set.

Naturally, we select reference sequences by evaluating the number of generated candidate motifs, ensuring the selected reference sequences generate a small number of candidate motifs. When we evaluate the number of candidate motifs generated from a tuple *T* of *h l*-mers, it is difficult to directly compute the number of common candidate motifs shared by all the *h l*-mers in *T*, denoted by *N*_1_. An alternative way is to compute the sum of the number of common candidate motifs shared by each pair of *l*-mers in *T*, denoted by *N*_2_ = Σ|*M*_*d*_(*x*_*i*_, *x*_*j*_)| for 1 ≤ *i* < *j* ≤ *h*. As shown in Fig. 5, *N*_1_ and *N*_2_ have the consistent tendency with the variation of the Hamming distance between the pairs of *l*-mers in *T*.

Furthermore, we use (1) and (2) to evaluate *N*_*r*_(*D*'), the number of candidate motifs generated from the reference sequence set *D*'. It is defined as the sum of the number of common candidate motifs shared by each pair of *l*-mers in *D*' (i.e., in every two sequences in *D*').

1 $$ {N}_r\left(D{\textstyle \mathit{\hbox{'}}}\right)\kern0.5em ={\displaystyle \sum_{1\le i<j\le \left|D{\textstyle \mathit{\hbox{'}}}\right|}}{N}_r\left({s}_i,{s}_j\right) $$

2 $$ {N}_r\left({s}_i,{s}_j\right)={\displaystyle \sum_{x{\in}_l{s}_i,x\hbox{'}{\in}_l{s}_j}\left|{M}_d\left(x,x\hbox{'}\right)\right|} $$

Our method of selecting reference sequences includes two steps. The first step is to compute the number of candidate motifs generated from every two sequences in *D*, in order to quickly evaluate the number of candidate motifs generated from the specified *k* reference sequences in the next step. The second step is to select *k* sequences from *D* to form the reference sequence set *D*' such that the number of candidate motifs generated from *D*' is as small as possible. In the following, we describe the two steps in detail.

### Step 1: computing the number of candidate motifs

We compute the number of candidate motifs generated from every two sequences *s*_*i*_ and *s*_*j*_ in *D* according to (2). For an *l*-mer *x* in *s*_*i*_ and an *l*-mer *x*' in *s*_*j*_, the number of their common candidate motifs |*M*_*d*_(*x*, *x*')| depends on their Hamming distance *d*_*H*_(*x*, *x*') [18]. The details about how to compute |*M*_*d*_(*x*, *x*')| are described in [18]. In implementing RefSelect, we store the values of |*M*_*d*_(*x*, *x*')| under different *d*_*H*_(*x*, *x*') in a table in advance. Once we know *d*_*H*_(*x*, *x*'), we can immediately get |*M*_*d*_(*x*, *x*')| by looking up the table in *O*(1) time.

Thus, the core operation of (2) is to compute the Hamming distance between every two *l*-mers *x* ∈_*l*_*s*_*i*_ and *x*' ∈_*l*_*s*_*j*_. For any two sequences of length *n*, we have *O*(*n*^2^) pairs of *l*-mers. A simple method is to traverse all these pairs of *l*-mers; for each pair of *l*-mers *x* and *x*', the Hamming distance can be computed in *O*(*l*) time by comparing the characters *x*[*i*] and *x*'[*i*] for 1 ≤ *i* ≤ *l*. The time complexity of this method is *O*(*ln*^2^).

**Fig. 2:**
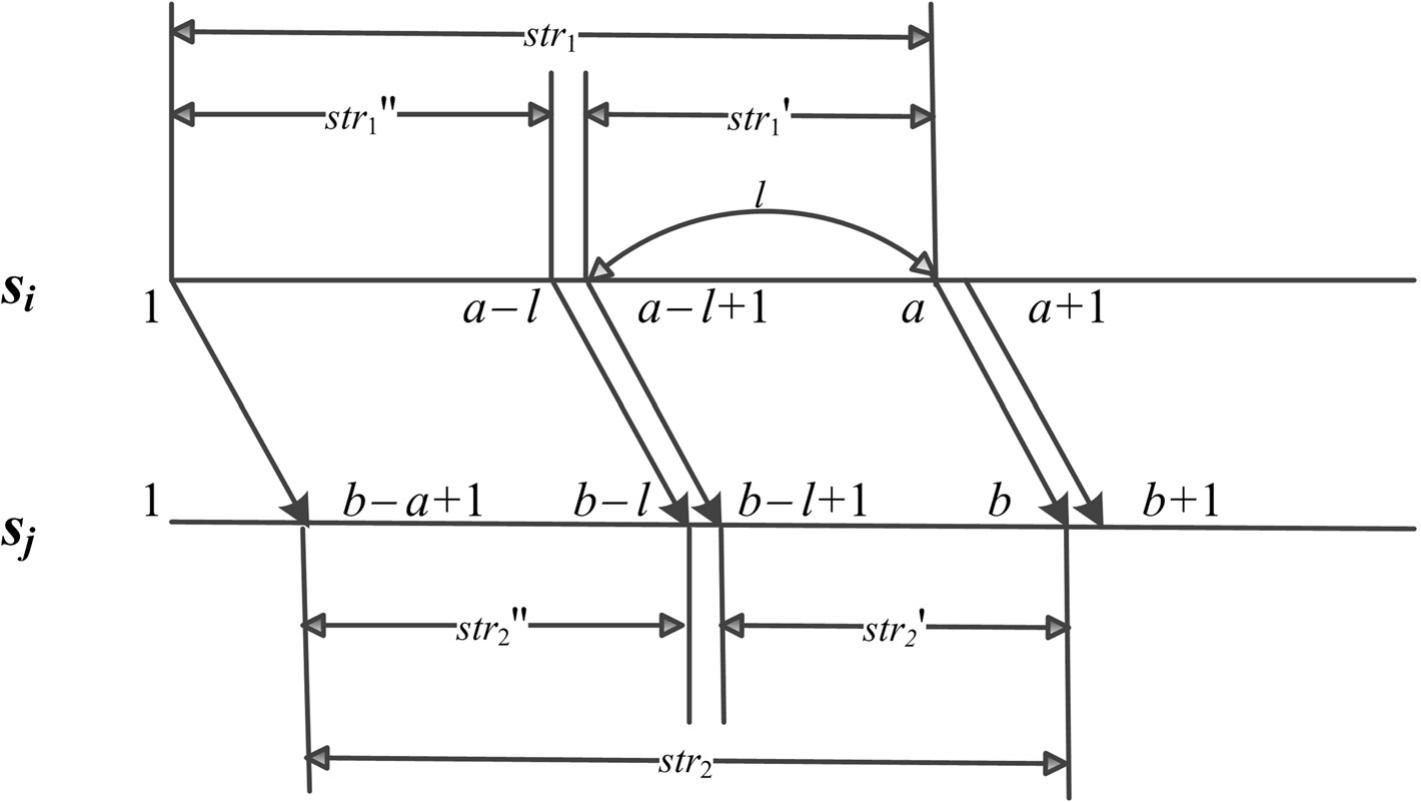
Illustration of fast computation of Hamming distance. This figure shows an example for explaining how to compute the Hamming distance between every pair of *l*-mers in two input sequences

We introduce a more efficient method to compute the Hamming distance between every pair of *l*-mers in *s*_*i*_ and *s*_*j*_. We fill an *n* × *n* matrix *M*, where the element in row *a* (1 ≤ *a* ≤ *n*) and column *b* (1 ≤ *b* ≤ *n*) is denoted by *M*[*a*, *b*]. Let *l*_*min*_ = *min*(*a*, *b*), *str*_1_ = *s*_*i*_[*a* – *l*_*min*_ + 1…*a*], *str*_2_ = *s*_*j*_[*b* – *l*_*min*_ *+* 1…*b*]; then, *M*[*a*, *b*] is the number of such position *i* that *str*_1_[*a* – *l*_*min*_ + *i*] = *str*_2_[*b* – *l*_*min*_ *+ i*] for 1 ≤ *i* ≤ *l*_*min*_, namely *M*[*a*, *b*] = *l*_*min*_ − *d*_*H*_(*str*_1_, *str*_2_). For example, in Fig. 2, *a* < *b*, *str*_1_ = *s*_*i*_[1…*a*], *str*_2_ = *s*_*j*_[*b* – *a* + 1…*b*], and then *M*[*a*, *b*] = *a* − *d*_*H*_(*s*_*i*_[1…*a*], *s*_*j*_[*b* – *a* + 1…*b*]), which denotes the number of positions where the two characters are identical in the alignment of *str*_1_ and *str*_2_.

In filling the matrix *M*, we initialize *M*[*a*, *b*] with 0 for the case of *min*(*a*, *b*) = 0, and obtain *M*[*a* + 1, *b* + 1] based on *M*[*a*, *b*]:

3 $$ M\left[a+1,b+1\right]=\left\{\begin{array}{c}\hfill M\left[a,b\right]+1,\kern0.5em \mathrm{if}\ {s}_i\left[a+1\right]={s}_j\left[b+1\right]\hfill \\ {}\hfill M\left[a,b\right],\kern5.5em \mathrm{otherwise}\hfill \end{array}\right., $$

where both *a* and *b* range from 0 to *n*.

With the matrix *M*, we use (4) to compute the Hamming distance between a pair of *l*-mers *str*_1_' and *str*_2_', where *str*_1_' = *s*_*i*_[*a* – *l* + 1…*a*] and *str*_2_' = *s*_*j*_[*b* – *l* + 1…*b*] are the *l*-mers at the position *a* (*a* ≥ *l*) of *s*_*i*_ and the position *b* (*b* ≥ *l*) of *s*_*j*_, respectively.

4 $$ {d}_H\left(st{r}_1\hbox{'},\kern0.5em st{r}_2\hbox{'}\right)\kern0.5em =\kern0.5em l\kern0.5em -\kern0.5em \left(M\left[a,b\right]\kern0.5em -\kern0.5em M\left[a\kern0.5em -\kern0.5em l,\kern0.5em b\kern0.5em -\kern0.5em l\right]\right) $$

Our method is mainly to fill the matrix *M*. That is, we need to compute *n*^2^ elements one by one for any two *n*-length sequences *s*_*i*_ and *s*_*j*_. In computing each element *M*[*a*, *b*] by (3) in *O*(1) time, we simultaneously compute the Hamming distance between the *l*-mer at position *a* (*a* ≥ *l*) of *s*_*i*_ and that at position *b* (*b* ≥ *l*) of *s*_*j*_ by (4) in *O*(1) time. Therefore, the time complexity of computing the Hamming distance for all pairs of *l*-mers in two sequences is reduced to *O*(*n*^2^).

### Step 2: selecting reference sequences

After getting the number of candidate motifs generated from every two sequences in *D*, we can evaluate the number of candidate motifs generated from a set of reference sequences according to (1). In this section, we introduce how to select a set of reference sequences that generates a small number of candidate motifs.

First, let us consider the exhaustive search strategy. For the data set *D* consisting of *t* sequences, after evaluating the number of candidate motifs for every possible *k* sequences in *D*, the exhaustive search regards the *k* sequences corresponding to the minimum number of candidate motifs as the reference sequences. Its time complexity is as follows.

$$ O\left(\left(\begin{array}{c}\hfill t\hfill \\ {}\hfill k\hfill \end{array}\right)\times \left(\begin{array}{c}\hfill k\hfill \\ {}\hfill 2\hfill \end{array}\right)\right) $$

Obviously, the running time of the exhaustive search grows dramatically with the increase of the number of input sequences *t* and the number of selected reference sequences *k*. A simple experiment can show that the exhaustive search is too time-consuming for large input: we set |Σ| = 4, *n* = 200, *q* = *t* = 200 and *k* = *t* × 5 % = 10, and the running time of the exhaustive search exceeds one day on personal computers.

In order to quickly select reference sequences, we convert the problem to graph clustering. The *t* sequences in *D* are taken as *t* nodes in a graph. The similarity between two nodes *s*_*i*_ and *s*_*j*_ is related to the number of candidate motifs generated from *s*_*i*_ and *s*_*j*_ (i.e., *N*_*r*_(*s*_*i*_*, s*_*j*_)). We hope that the nodes corresponding to the reference sequences with small number of candidate motifs form a dense subgraph, and they belong to the same cluster after graph clustering. We use the MCL algorithm [25] to complete clustering, and set the inflation parameter as 1.8 following [26]. We describe the details involved in clustering as follows.

#### Similarity measure

We design the similarity of two nodes (sequences) *s*_*i*_ and *s*_*j*_ based on *N*_*r*_(*s*_*i*_*, s*_*j*_). Simultaneously, we consider the following two factors.

First, we further increase the effect of the pairs of *l*-mers in *s*_*i*_ and *s*_*j*_ with small Hamming distance on the total number of candidate motifs generated from *s*_*i*_ and *s*_*j*_. By doing this, it is helpful for the clustering process to distinguish different reference sequence sets that lead to different number of candidate motifs. Specifically, we use (5) instead of (2) to evaluate the number of candidate motifs generated from two sequences *s*_*i*_ and *s*_*j*_.

5 $$ {N}_r\hbox{'}\left({s}_i,{s}_j\right)={\displaystyle \sum_{x{\in}_l{s}_i,x\hbox{'}{\in}_l{s}_j}\frac{\left|{M}_d\left(x,x\hbox{'}\right)\right|}{d_H\left(x,x\hbox{'}\right)+1}} $$

Second, we aim to put a set of sequences *D*' to the same cluster such that every two sequences *s*_*i*_ and *s*_*j*_ in *D*' generate a small number of candidate motifs. So, we should ensure that *s*_*i*_ and *s*_*j*_ have a larger similarity when they generate a smaller number of candidate motifs. Finally, we compute the similarity of *s*_*i*_ and *s*_*j*_ as follows:

6 $$ sim\left({s}_i,{s}_j\right)=\frac{1}{N_r\hbox{'}\left({s}_i,{s}_j\right)}\times \underset{1\le i<j\le t}{ \max }{N}_r\hbox{'}\left({s}_i,{s}_j\right). $$

#### Cluster refinement

The clustering process may produce more than one cluster, and there may not be exact *k* nodes in each cluster. We refine each obtained cluster *C* in order to get a set of *k* reference sequences. Then, we sort the sets of reference sequences and output the set with the highest score.

For the cluster *C* with only one node, we take it as an invalid cluster, since the node in *C* has a low similarity with other nodes. For the cluster *C* with two or more nodes, it corresponds to three cases: (a) there are exact *k* nodes in *C*; (b) there are more than *k* nodes in *C*; (c) there are less than *k* nodes in *C*. For Case (a), we can get the reference sequence set *D*' directly by using the *k* sequences in *C*. Next, we introduce how to refine *C* under Cases (b) and (c).

For Case (b), we use greedy strategy to select *k* sequences from *C* (|*C*| > *k*) to form *D*'. First, we initialize *D*' with {*s*_*a*_, *s*_*b*_} such that *sim*(*s*_*a*_, *s*_*b*_) = max{*sim*(*s*_*i*_, *s*_*j*_)} for all *s*_*i*_, *s*_*j*_ ∈ *C* and *s*_*i*_ ≠ *s*_*j*_. Then, we repeatedly choose a node *s*_*r*_ satisfying (7) from *C* − *D*' and add it to *D*' until |*D*'| = *k*.

7 $$ {s}_r=\underset{s_i\in C-D\hbox{'}}{ \arg \max }{\displaystyle \sum_{s_j\in D\hbox{'}}sim\left({s}_i,\ {s}_j\right)} $$

For Case (c), we use the similar method to choose *k* − |*C*| nodes from *D* − *C*, and add them to *C* to form *D*'. First, *D*' is initialized with *C*. Then, we repeatedly choose a node *s*_*r*_ satisfying (8) from *D* − *D*' and add it to *D*' until |*D*'| = *k*.

8 $$ {s}_r=\underset{s_i\in D-D\hbox{'}}{ \arg \max }{\displaystyle \sum_{s_j\in D\hbox{'}}sim\left({s}_i,\ {s}_j\right)} $$

**Fig. 3:**
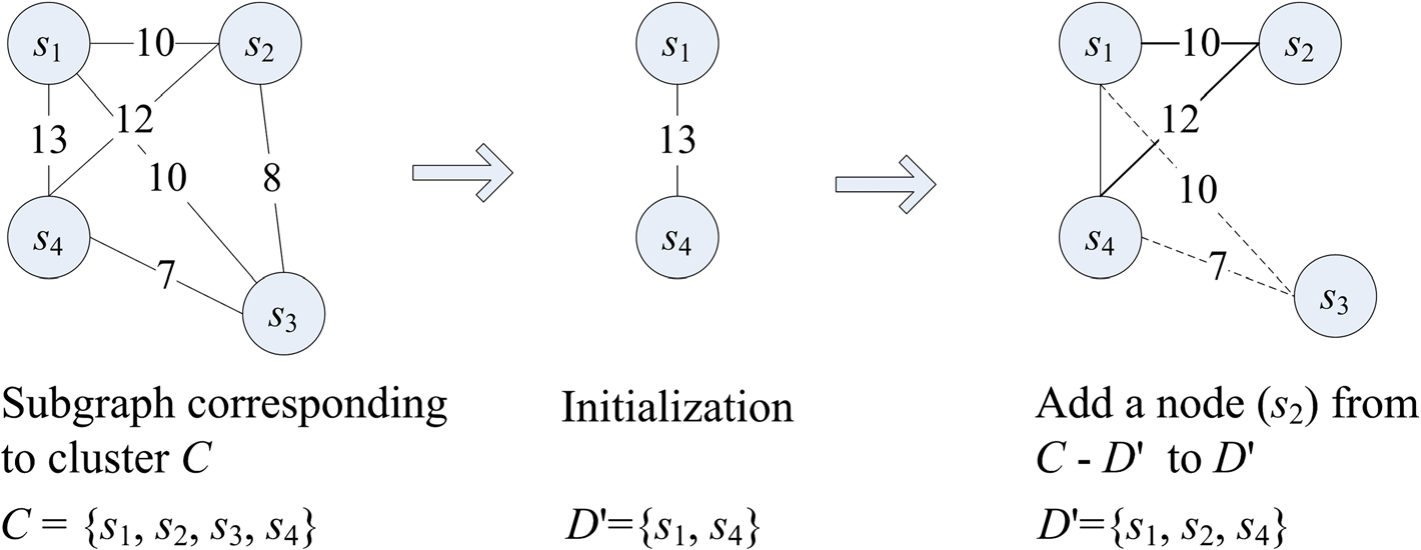
An example of cluster refinement for |*C*| > *k.* This figure shows an example of cluster refinement in the case that there are more than *k* nodes in the cluster *C*

**Fig. 4:**
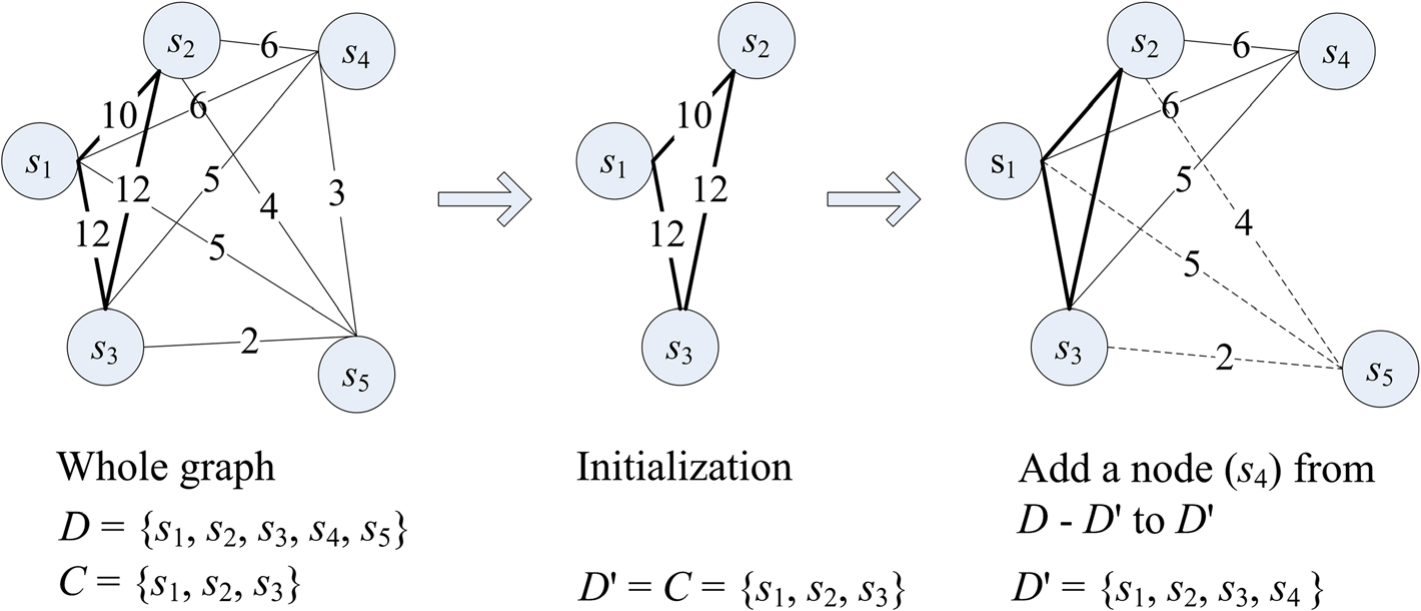
An example of cluster refinement for |*C*| < *k*. This figure shows an example of cluster refinement in the case that there are less than *k* nodes in the cluster *C*

Figures 3 and 4 show examples under Case (b) with *k* = 3 and Case (c) with *k* = 4, respectively. Differences between the two cases are: in Case (b), we get *D*' by selecting reference sequences (nodes) from the sub-graph corresponding to the cluster *C*; while in Case (c), we get *D*' by selecting reference sequences (nodes) from the whole graph and adding them to *C*.

We describe how to refine a cluster *C* in Algorithm 1. Because the process that we select reference sequences by using greedy strategy is similar to the Prim algorithm for computing minimum spanning tree, the time complexity of Algorithm 1 is *O*(|*C*|^2^lg|*C*|) and *O*((*t* − |*C*|)^2^lg(*t* − |*C*|)) under Case (b) and Case (c), respectively.

After cluster refinement, if we obtain more than one reference sequence set *D*', we score each *D*' by (9), and then output the *D*' with the highest score.

9 $$ score\left(D\hbox{'}\right)={\displaystyle \sum_{s_i\in D\hbox{'},{s}_j\in D\hbox{'},i\ne j}sim\left({s}_i,{s}_j\right)} $$

### Whole algorithm

This section gives the whole algorithm of RefSelect.

In line 1 of the pseudocode, we initialize *D*' with an empty set. Lines 2 to 5, corresponding to the first step of RefSelect, compute the similarity of any two nodes (sequences). The core operation of this step is to compute the Hamming distance from all *l*-mers in *s*_*i*_ to all *l*-mers in *s*_*j*_ in *O*(*n*^2^) time, for any two sequences *s*_*i*_ and *s*_*j*_ in *D*. Therefore, the time complexity of this step is:

$$ O\left(\left(\begin{array}{c}\hfill t\hfill \\ {}\hfill 2\hfill \end{array}\right)\times {n}^2\right). $$

Lines 6 to 10, corresponding to the second step of RefSelect, cluster the *t* sequences in *D* by using the MCL algorithm and refine each obtained cluster. The time complexity of clustering is *O*(*t*^3^). The time complexity of refining clusters is negligible with respect to the time complexity of the first step. So, the time complexity of RefSelect is:

$$ O\left(\left(\begin{array}{c}\hfill t\hfill \\ {}\hfill 2\hfill \end{array}\right)\times {n}^2+{t}^3\right). $$

In executing RefSelect, we need *O*(*tn*) space to store the input sequence set *D*, *O*(*n*^2^) space to store the matrix *M* for computing Hamming distance, and *O*(*t*^2^) space to store the similarity matrix of *t* input sequences. So, the space complexity of RefSelect is *O*(*tn* + *n*^2^ + *t*^2^).

### Parallel implementation

To efficiently deal with large data sets, we can further accelerate RefSelect by using parallel processing. RefSelect consists of two steps, and we mainly use parallel processing for the first step. The reasons are (1) the first step is the bottleneck of the whole RefSelect algorithm in running time as shown in Table 6, and (2) the first step is easy to parallelize, because it repeatedly calculates the number of candidate motifs generated from two sequences *s*_*i*_ and *s*_*j*_ in *D* and each calculation is independent of the others.

We implement the parallel version of RefSelect by using OpenMP [27, 28], which provides a flexible programming model for shared memory architectures and allows to add parallelism into serial codes easily by using one or several OpenMP directives. The pseudocode is shown in Algorithm 3, where we add an OpenMP “for” directive before the inner iterations of the first step to split parallel iteration spaces across threads.

The reason why we add the directive before the inner iterations (line 4) rather than the outer iterations (line 2) is for the consideration of load balancing among threads. Note that, the number of inner iterations is not fixed for each outer iteration. If we add the directive before the outer iterations, the smaller the value of *i*, the more computational work will be needed by the thread processing the *i*th outer iteration.

## Results and discussion

### Analysis of effect of Hamming distance on candidate motif number

First, we consider the case of two *l*-mers *x* and *x*', and analyze the effect of their Hamming distance *d*_*H*_(*x*, *x*') on the number of their common candidate motifs |*M*_*d*_(*x*, *x*')|. Tables 2 and 3 give the values of |*M*_*d*_(*x*, *x*')| with *d*_*H*_(*x*, *x*') varying from 0 to 2*d* under the DNA and protein data, respectively. In both tables, the values are obtained by using two challenging PMS problem instances. We can find that |*M*_*d*_(*x*, *x*')| increases with the decrease of *d*_*H*_(*x*, *x*') for both the DNA and protein data.

**Table 2:** The effect of Hamming distance on the candidate motif number and occurrence probability for a pair of *l*-mers under the DNA data

*i =* *d*_*H*_(*x, x'*)	(13, 4)		(15, 5)		(17, 6)		(19, 7)	
	|*M*_*d*_(*x*, *x*')|	*p*_*i*_	|*M*_*d*_(*x*, *x*')|	*p*_*i*_	|*M*_*d*_(*x*, *x*')|	*p*_*i*_	|*M*_*d*_(*x*, *x*')|	*p*_*i*_
14	-	-	-	-	-	-	3.4 × 10^3^	2.0 × 10^−1^
13	-	-	-	-	-	-	2.7 × 10^4^	1.6 × 10^−1^
12	-	-	-	-	9.2 × 10^2^	1.9 × 10^−1^	1.1 × 10^5^	9.7 × 10^−2^
11	-	-	-	-	6.5 × 10^3^	1.3 × 10^−1^	3.2 × 10^5^	4.9 × 10^−2^
10	-	-	2.5 × 10^2^	1.7 × 10^−1^	2.4 × 10^4^	6.7 × 10^−2^	7.5 × 10^5^	2.0 × 10^−2^
9	-	-	1.5 × 10^3^	9.2 × 10^−2^	6.4 × 10^4^	2.8 × 10^−2^	1.6 × 10^6^	6.6 × 10^−3^
8	7.0 × 10^1^	1.3 × 10^−1^	5.0 × 10^3^	3.9 × 10^−2^	1.4 × 10^5^	9.3 × 10^−3^	2.9 × 10^6^	1.8 × 10^−3^
7	3.5 × 10^2^	5.6 × 10^−2^	1.3 × 10^4^	1.3 × 10^−2^	2.8 × 10^5^	2.5 × 10^−3^	5.0 × 10^6^	4.0 × 10^−4^
6	1.1 × 10^3^	1.9 × 10^−2^	2.7 × 10^4^	3.4 × 10^−3^	5.0 × 10^5^	5.3 × 10^−4^	8.2 × 10^6^	7.2 × 10^−5^
5	2.5 × 10^3^	4.7 × 10^−3^	5.1 × 10^4^	6.8 × 10^−4^	8.5 × 10^5^	8.8 × 10^−5^	1.3 × 10^7^	1.0 × 10^−5^
4	5.2 × 10^3^	8.6 × 10^−4^	9.0 × 10^4^	1.0 × 10^−4^	1.4 × 10^6^	1.1 × 10^−5^	**2.0 × 10** ^**7**^	**1.1 × 10** ^**−6**^
3	9.3 × 10^3^	1.2 × 10^−4^	1.5 × 10^5^	1.1 × 10^−5^	**2.1 × 10** ^**6**^	**1.1 × 10** ^**−6**^	**3.0 × 10** ^**7**^	**9.5 × 10** ^**−8**^
2	1.7 × 10^4^	1.1 × 10^−5^	**2.5 × 10** ^**5**^	**8.8 × 10** ^**−7**^	**3.4 × 10** ^**6**^	**7.1 × 10** ^**−8**^	**4.6 × 10** ^**7**^	**5.6 × 10** ^**−9**^
1	**2.6 × 10** ^**4**^	**5.8 × 10** ^**−7**^	**3.7 × 10** ^**5**^	**4.2 × 10** ^**−8**^	**4.9 × 10** ^**6**^	**3.0 × 10** ^**−9**^	**6.4 × 10** ^**7**^	**2.1 × 10** ^**−10**^
0	**6.6 × 10** ^**4**^	**1.5 × 10** ^**−8**^	**8.5 × 10** ^**5**^	**9.3 × 10** ^**−10**^	**1.1 × 10** ^**7**^	**5.8 × 10** ^**−11**^	**1.3 × 10** ^**8**^	**3.6 × 10** ^**−12**^

**Table 3:** The effect of Hamming distance on the candidate motif number and occurrence probability for a pair of *l*-mers under the protein data

*i* *= d*_*H*_(*x, x'*)	(13, 6)		(15, 7)		(17, 8)		(19, 9)	
	|*M*_*d*_(*x*, *x*')|	*p*_*i*_	|*M*_*d*_(*x*, *x*')|	*p*_*i*_	|*M*_*d*_(*x*, *x*')|	*p*_*i*_	|*M*_*d*_(*x*, *x*')|	*p*_*i*_
18	-	-	-	-	-	-	4.9 × 10^4^	3.8 × 10^−1^
17	-	-	-	-	-	-	4.0 × 10^6^	1.8 × 10^−1^
16	-	-	-	-	1.3 × 10^4^	3.7 × 10^−1^	1.4 × 10^8^	5.3 × 10^−2^
15	-	-	-	-	9.4 × 10^5^	1.6 × 10^−1^	2.6 × 10^9^	1.1 × 10^−2^
14	-	-	3.4 × 10^3^	3.7 × 10^−1^	2.8 × 10^7^	4.1 × 10^−2^	3.1 × 10^10^	1.8 × 10^−3^
13	-	-	2.2 × 10^5^	1.4 × 10^−1^	4.6 × 10^8^	7.6 × 10^−3^	2.4 × 10^11^	2.2 × 10^−4^
12	9.2 × 10^2^	3.5 × 10^−1^	5.6 × 10^6^	3.1 × 10^−2^	4.5 × 10^9^	1.0 × 10^−3^	1.3 × 10^12^	2.1 × 10^−5^
11	5.1 × 10^4^	1.1 × 10^−1^	7.6 × 10^7^	4.9 × 10^−3^	2.9 × 10^10^	1.1 × 10^−4^	**5.4 × 10** ^**12**^	**1.7 × 10** ^**−6**^
10	1.1 × 10^6^	2.1 × 10^−2^	6.0 × 10^8^	5.6 × 10^−4^	1.3 × 10^11^	9.1 × 10^−6^	**1.8 × 10** ^**13**^	**1.1 × 10** ^**−7**^
9	1.2 × 10^7^	2.8 × 10^−3^	3.0 × 10^9^	4.9 × 10^−5^	**4.4 × 10** ^**11**^	**6.0 × 10** ^**−7**^	**5.0 × 10** ^**13**^	**5.7 × 10** ^**−9**^
8	7.0 × 10^7^	2.7 × 10^−4^	1.1 × 10^10^	3.3 × 10^−6^	**1.3 × 10** ^**12**^	**3.2 × 10** ^**−8**^	**1.2 × 10** ^**14**^	**2.5 × 10** ^**−10**^
7	2.7 × 10^8^	1.9 × 10^−5^	**3.2 × 10** ^**10**^	**1.8 × 10** ^**−7**^	**3.1 × 10** ^**12**^	**1.3 × 10** ^**−9**^	**2.8 × 10** ^**14**^	**8.6 × 10** ^**−12**^
6	**8.3 × 10** ^**8**^	**9.9 × 10** ^**−7**^	**8.0 × 10** ^**10**^	**7.2 × 10** ^**−9**^	**7.1 × 10** ^**12**^	**4.4 × 10** ^**−11**^	**5.9 × 10** ^**14**^	**2.4 × 10** ^**−13**^
5	**2.1 × 10** ^**9**^	**3.9 × 10** ^**−8**^	**1.8 × 10** ^**11**^	**2.3 × 10** ^**−10**^	**1.5 × 10** ^**13**^	**1.2 × 10** ^**−12**^	**1.2 × 10** ^**15**^	**5.5 × 10** ^**−15**^
4	**4.7 × 10** ^**9**^	**1.1 × 10** ^**−9**^	**3.8 × 10** ^**11**^	**5.4 × 10** ^**−12**^	**3.0 × 10** ^**13**^	**2.4 × 10** ^**−14**^	**2.3 × 10** ^**15**^	**9.6 × 10** ^**−17**^
3	**9.8 × 10** ^**9**^	**2.4 × 10** ^**−11**^	**7.5 × 10** ^**11**^	**9.5 × 10** ^**−14**^	**5.7 × 10** ^**13**^	**3.6 × 10** ^**−16**^	**4.3 × 10** ^**15**^	**1.3 × 10** ^**−18**^
2	**2.0 × 10** ^**10**^	**3.4 × 10** ^**−13**^	**1.5 × 10** ^**12**^	**1.2 × 10** ^**−15**^	**1.1 × 10** ^**14**^	**3.8 × 10** ^**−18**^	**8.0 × 10** ^**15**^	**1.2 × 10** ^**−20**^
1	**4.1 × 10** ^**10**^	**3.0 × 10** ^**−15**^	**2.9 × 10** ^**12**^	**8.7 × 10** ^**−18**^	**2.1 × 10** ^**14**^	**2.5 × 10** ^**−20**^	**1.6 × 10** ^**16**^	**6.9 × 10** ^**−23**^
0	**8.4 × 10** ^**10**^	**1.2 × 10** ^**−17**^	**6.0 × 10** ^**12**^	**3.1 × 10** ^**−20**^	**4.3 × 10** ^**14**^	**7.6 × 10** ^**−23**^	**3.1 × 10** ^**16**^	**1.9 × 10** ^**−25**^

**Fig. 5:**
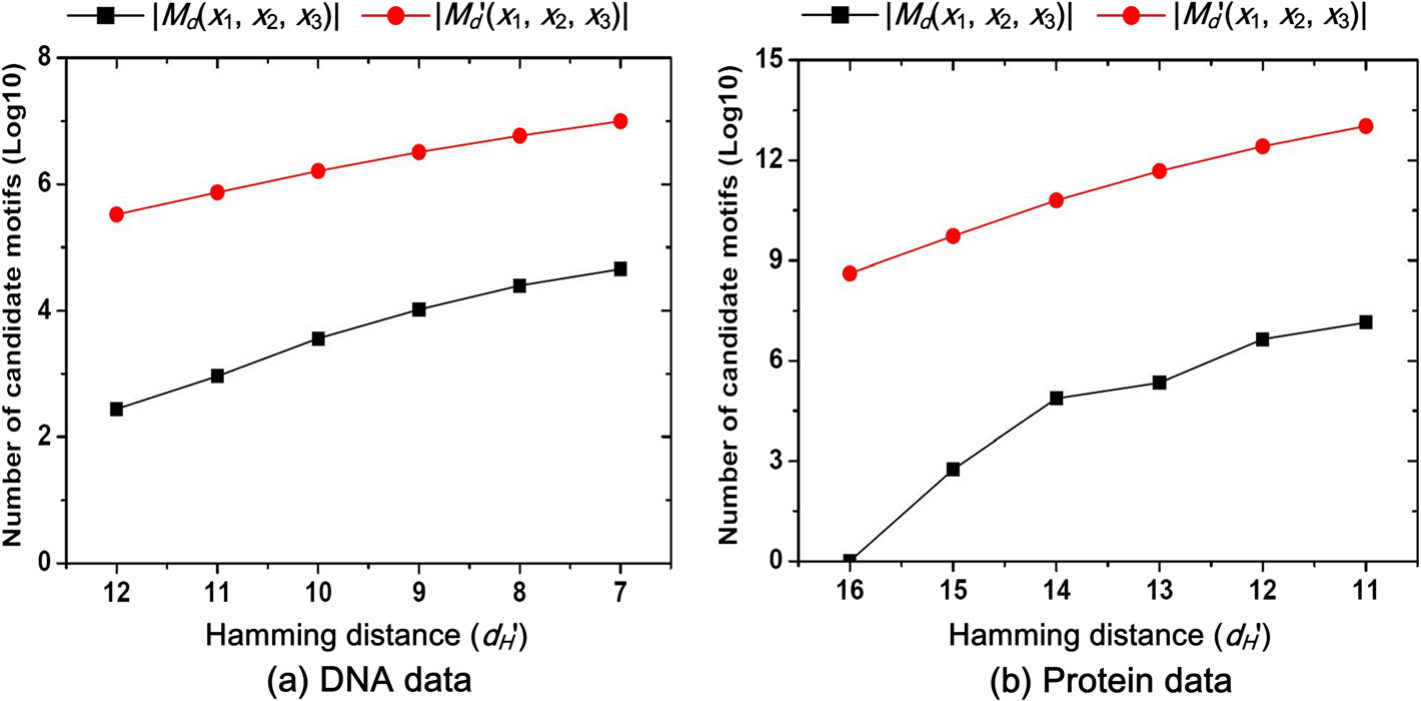
The effect of Hamming distance on the number of candidate motifs for three *l*-mers. This figure shows the effect of Hamming distance on the number of candidate motifs for three *l*-mers *x*_1_, *x*_2_ and *x*_3_. The used (*l*, *d*) is set as (19, 7) and (19, 9) under the DNA data and protein data, respectively. We fix *d*_*H*_(*x*_1_, *x*_2_) = 2*d* − 2 and vary *d*_*H*_' = (*d*_*H*_(*x*_1_, *x*_3_) + *d*_*H*_(*x*_2_, *x*_3_))/2 from 2*d* − 2 to 2*d* − 7. |*M*_*d*_(*x*_1_, *x*_2_, *x*_3_)| is the number of common candidate motifs shared by the three *l*-mers. |*M*_*d*_'(*x*_1_, *x*_2_, *x*_3_)| = |*M*_*d*_(*x*_1_, *x*_2_)| + |*M*_*d*_(*x*_1_, *x*_3_)| + |*M*_*d*_(*x*_2_, *x*_3_)| is the sum of the number of common candidate motifs shared by each pair of *l*-mers. **a** it is for the DNA data. **b** it is for the protein data

Second, we consider the case of *h* (*h* > 2) *l*-mers containing pairs of *l*-mers with different Hamming distance, and analyze the effect of Hamming distance on the number of common candidate motifs shared by the *h l*-mers. In our example, we set *h* as 3 and the three *l*-mers *x*_1_, *x*_2_ and *x*_3_ can form three pairs of *l*-mers; then, we fix *d*_*H*_(*x*_1_, *x*_2_) = 2*d* − 2 and vary *d*_*H*_' = (*d*_*H*_(*x*_1_, *x*_3_) + *d*_*H*_(*x*_2_, *x*_3_))/2 from 2*d* − 2 to 2*d* − 7. Figure 5(a) and (b) give the tendency of the number of common candidate motifs |*M*_*d*_(*x*_1_, *x*_2_, *x*_3_)| in contact with the decrease of *d*_*H*_' on (19, 7) problem instance for the DNA data and (19, 9) problem instance for the protein data, respectively. The y-axis is in log-scale. We can see that no matter for the DNA or protein data, |*M*_*d*_(*x*_1_, *x*_2_, *x*_3_)| increases with the decrease of *d*_*H*_*'*. In other words, when *h* (*h* > 2) *l*-mers contain some pairs of *l*-mers with a relatively small Hamming distance, they generate a relatively large number of candidate motifs. Also, the tendency of |*M*_*d*_'(*x*_1_, *x*_2_, *x*_3_)| = |*M*_*d*_(*x*_1_, *x*_2_)| + |*M*_*d*_(*x*_1_, *x*_3_)| + |*M*_*d*_(*x*_2_, *x*_3_)| is given in Fig. 5. Both |*M*_*d*_'(*x*_1_, *x*_2_, *x*_3_)| and |*M*_*d*_(*x*_1_, *x*_2_, *x*_3_)| increase with the decrease of *d*_*H*_', namely they have the consistent tendency.

In addition, Tables 2 and 3 also give the probability that the Hamming distance between two random *l*-mers *x* and *x*' is *i* (0 ≤ *i* ≤ 2*d*), denoted by *p*_*i*_ and calculated by (10). As seen from the tables, *p*_*i*_ decreases with the decrease of the Hamming distance *i*.

10 $$ {p}_i=\left(\kern1em \begin{array}{c}l\kern1em \\ {}\kern1em i\end{array}\kern1em \right)\frac{{\left(\left|\Sigma \right|-1\right)}^i}{{\left|\Sigma \right|}^l} $$

For two *n*-length sequences *s*_*i*_ and *s*_*j*_, let *E*_*i*_ denote the expected number of the pair of *l*-mers *x* ∈_*l*_*s*_*i*_ and *x*' ∈_*l*_*s*_*j*_ with *d*_*H*_(*x*, *x*') = *i*. It is calculated by (11). The bold values in Tables 2 and 3 indicate that *E*_*i*_ is less than 1 in the case of *n* = 600.

11 $$ {E}_i={\left(n-l + 1\right)}^2\times {p}_i $$

Although they rarely occur in two sequences, some pairs of *l*-mers with *E*_*i*_ < 1 are usually contained in the whole data set. The reasons are: first, the whole data set can form multiple pairs of sequences, which increases the probability of the occurrence of the pairs of *l*-mers with Hamming distance *i*; second, the conservation of motifs makes some highly similar motif instances form some pairs of *l*-mers with *E*_*i*_ < 1.

From the tables, when *E*_*i*_ is less than 1, the value of |*M*_*d*_(*x*, *x*')| is relatively large, especially for the protein data. Thus, the more pairs of *l*-mers with *E*_*i*_ < 1 in the reference sequence set, the more candidate motifs generated by the algorithms.

### Results on practical time improvement of PMS algorithms

In this section we check the validity of RefSelect as follows. First, we use RefSelect to select *k* reference sequences from the given *t* input sequences, and adjust the order of the *t* input sequences by preposing the *k* sequences; RefSelect is implemented in C++ and its running time is denoted by *T*_*rs*_. Second, we test pattern-driven PMS algorithms on the input sequences of original order and that of new order, obtaining the running time *T*_1_ and *T*_2_, respectively. Finally, we compare *T*_1_ with *T*_*rs*_ + *T*_2_.

Three pattern-driven PMS algorithms qPMS7 [19], TravStrR [20] and PMS8 [23] are chosen to participate in the test. They are all newly proposed algorithms and outperform the previous exact algorithms on challenging instances. Notice that qPMS9 [24] is also a newly proposed PMS algorithm with good time performance; we do not choose it as a tested algorithm and related discussion is given in the applicability of RefSelect section. All the tested algorithms are executed on a 2.67 GHz single core and a 4 Gbyte Memory, except for PMS8, which is executed on a 16-core platform in solving the (21, 10) and (23, 11) instances of the protein data.

In the experiments, we generate data sets following [5]. First, we randomly generate *t* sequences of length *n* and a motif *m* of length *l*, and randomly choose *q* (0 < *q* ≤ *t*) out of the *t* sequences; then, for each of the *q* sequences, we generate a random motif instance *m*' that differs from *m* in at most *d* positions, and implant *m*' into a random position of the sequence. For each specific test instance, we generate five data sets to get an average result.

First, we fix *t* = 20, *n* = 600 and *q* = 20, and give in Tables 4 and 5 the results on challenging instances of the protein and DNA data, respectively. For qPMS7 and TravStrR, *k* is set as 2, while for PMS8 *k* is set dynamically under different (*l*, *d*) instances according to [23]. From this experiment, we find that:

**Table 4:** Running time and speedup for the protein data

(*l*, *d*)		qPMS7			TravStrR			PMS8		
		*T*_1_	*T*_*rs*_ + *T*_2_	*speedup*	*T*_1_	*T*_*rs*_ + *T*_2_	*speedup*	*T*_1_	*T*_*rs*_ + *T*_2_	*speedup*
(15, 7)	min	93.00s	85.00s	6.58	5.80s	5.63 s	1.00	2.21 s	3.64 s	1.89
	ave	722.40s	109.80s		7.51 s	7.49 s		288.33 s	178.43 s	
	max	2596.00s	122.00s		8.89 s	8.74 s		1126.80s	544.00s	
(17, 8)	min	131.00s	120.00s	7.68	11.88 s	8.98 s	4.03	390.13 s	8.52 s	5.85
	ave	1204.40s	156.80s		76.98 s	19.10s		1647.89 s	281.83 s	
	max	3742.00s	214.00s		207.32 s	54.01 s		3092.82 s	719.78 s	
(19, 9)	min	820.00s	140.00s	86.22	11.58 s	11.25 s	76.38	189.07 s	17.11 s	2.21
	ave	18502.20s	214.60s		1591.67 s	20.84 s		14619.67 s	6629.83 s	
	max	37121.00s	287.00s		4355.44 s	30.23 s		53376.12 s	17235.40s	
(21, 10)	min	191.00s	161.00s	>118.94	22.47 s	22.28 s	61.63	18268.00s	3022.00s	>9.65
	ave	-o	671.40s		1745.09 s	24.25 s		-o	17905.40s	
	max	-o	1783.00s		6453.27 s	53.06 s		-o	31054.00s	
(23, 11)	min	53978.00s	242.00s	>34.77	44.30s	12.19 s	15.56	6327.12 s	1608.23 s	>12.89
	ave	-o	4970.00s		3033.99 s	195.04 s		-o	13409.80s	
	max	-o	17141.00s		10909.72 s	378.31 s		-o	50421.00s	

s: seconds; −o:over 48 hours; *T*_1_ and *T*_2_: running time of a PMS algorithm on the input sequences of original order and new order; *T*_*rs*_: running time of RefSelect; min, ave and max: the minimum, average and maximum running time on five data sets; speedup: average *T*_1_/average *T*_*rs*_ + *T*_2_.

**Table 5:** Running time and speedup for the DNA data

(*l*, *d*)		qPMS7			TravStrR			PMS8		
		*T*_1_	*T*_*rs*_ + *T*_2_	*speedup*	*T*_1_	*T*_*rs*_ + *T*_2_	*speedup*	*T*_1_	*T*_*rs*_ + *T*_2_	*speedup*
(15, 5)	min	149.00s	128.00s	1.10	68.34 s	64.46 s	1.03	38.51 s	37.73 s	1.04
	ave	173.00s	157.67 s		73.84 s	71.61 s		65.43 s	63.21 s	
	max	220.00s	196.00s		91.96 s	85.52 s		113.16 s	106.54 s	
(17, 6)	min	557.00s	504.00s	1.08	230.41 s	185.54 s	1.03	244.95 s	216.00s	1.04
	ave	660.40s	611.80s		263.39 s	255.38 s		387.80s	371.42 s	
	max	884.00s	756.00s		322.78 s	319.74 s		601.29 s	601.20s	
(19, 7)	min	2520.00s	2357.00s	1.06	1009.06 s	970.67 s	1.05	1105.88 s	1092.14 s	1.04
	ave	2911.00s	2737.20s		1116.18 s	1060.56 s		1834.32 s	1753.06 s	
	max	3846.00s	3553.00s		1281.81 s	1264.79 s		3064.09 s	2811.09 s	
(21, 8)	min	12144.00s	11343.00s	1.03	3403.84 s	3168.97 s	1.00	7997.65	7896.77 s	1.13
	ave	12791.40s	12444.00s		4377.85 s	4357.19 s		12494.79 s	11102.56 s	
	max	13701.00s	14029.00s		5306.58 s	5301.84 s		14243.2 s	11395.31 s	
(23, 9)	min	61512.00s	61561.00s	1.07	14456.36 s	14184.14 s	1.00	23984.70s	22081.70s	1.11
	ave	68741.60s	64022.60s		18234.85 s	18157.41 s		38705.98 s	34847.10s	
	max	77427.00s	68709.00s		21315.48 s	22094.73 s		61160.00s	61278.10s	

s: seconds; −o:over 48 hours; *T*_1_ and *T*_2_: running time of a PMS algorithm on the input sequences of original order and new order; *T*_*rs*_: running time of RefSelect; min, average and max: the minimum, average and maximum running time on five data sets; speedup: average *T*_1_/average *T*_*rs*_ + *T*_2_.

1. The running time of RefSelect on each of these data sets is less than one second, which is nearly negligible and not listed in Tables 4 and 5.
2. RefSelect can make the tested algorithms solve the PMS problem steadily in an efficient way. For example, for the (19, 9) problem instance in Table 4, the minimum and maximum running time of qPMS7 are reduced to 140.00s and 287.00s from 820.00s and 37121.00s after using the reference sequences selected by RefSelect.
3. The speedup on the protein data is significantly larger than that on the DNA data. We give the explanation by using Tables 2 and 3. The fact that Pattern-driven PMS algorithms sometimes show poor performance is mainly caused by the pairs of *l*-mers with *E*_*i*_ < 1 in the reference sequences; these *l*-mers can generate more candidate motifs. The larger the difference between the number of candidate motifs for *E*_*i*_ < 1 and that for *E*_*i*_ ≥ 1, the larger speedup can be achieved. As can be seen from Tables 2 and 3, the difference on the protein data (large alphabet) is significantly larger than that on the DNA data (small alphabet).
4. Larger speedup is achieved on larger (*l*, *d*) instances for the protein data. This can also be explained by *E*_*i*_. That is, as shown in Tables 2 and 3, the difference between the number of candidate motifs for *E*_*i*_ < 1 and that for *E*_*i*_ ≥ 1 increases with the increase of *l* and *d*.

**Fig. 6:**
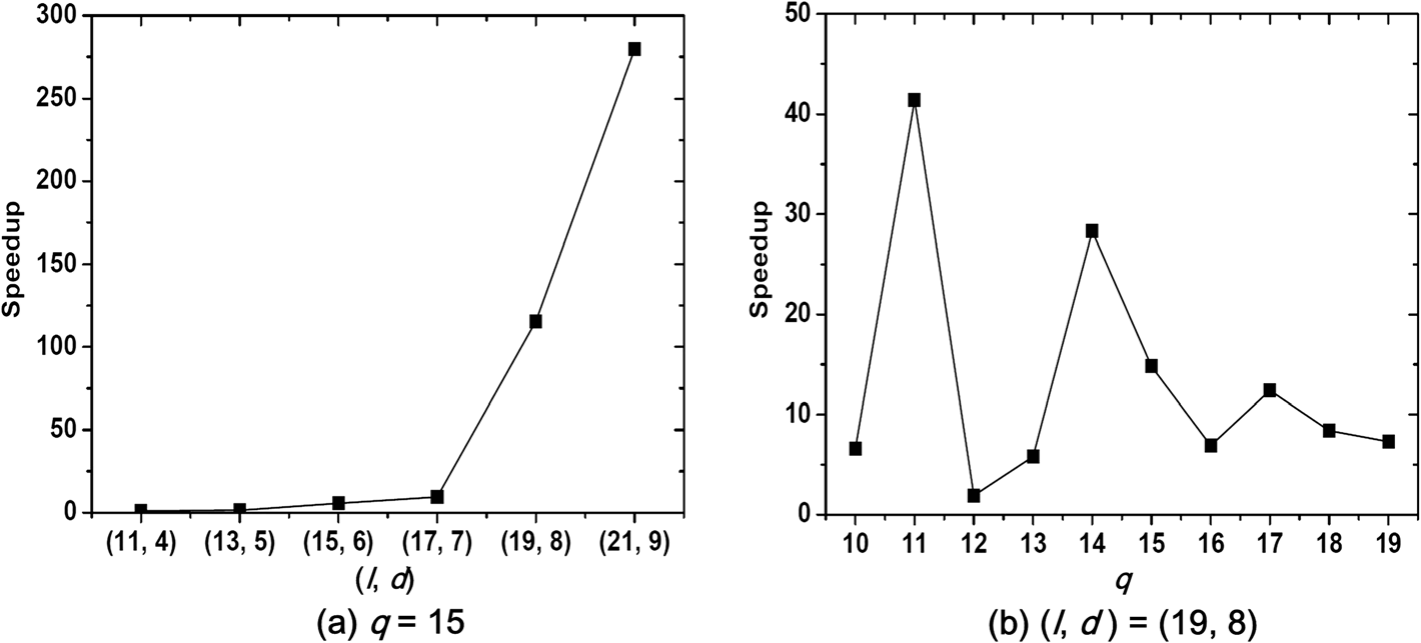
The speedup brought by RefSelect for qPMS7 in the case of *q* < *t*. This figure shows the speedup brought by RefSelect for qPMS7 in the case of *q* < *t*. **a** we fix *q* = 15 and vary (*l*, *d*) from (11, 4) to (21, 9). **b** we fix (*l*, *d*) = (19, 8) and vary *q* from 10 to 19

Second, we discuss the case of *q* < *t* by fixing |Σ| = 20 (protein data), *t* = 20 and *n* = 600. In the above three algorithms, we only choose qPMS7 as the tested algorithm because PMS8 cannot solve the PMS problem with *q* < *t* and TravStrR usually quits unexpectedly in our test environment. We select *t* – *q* + 2 reference sequences for qPMS7, and test it by using the same (*l*, *d*) instances with [19]. On the one hand, we set *q* as 15, and test qPMS7 on different (*l*, *d*) instances; as shown in Fig. 6(a), RefSelect makes qPMS7 perform better and the speedup increases with the increase of *l* and *d*. On the other hand, we fix (*l*, *d*) = (19, 8) and test qPMS7 by varying *q* from 10 to 19; as shown in Fig. 6(b), RefSelect can effectively accelerate qPMS7 under different *q*.

**Fig. 7:**
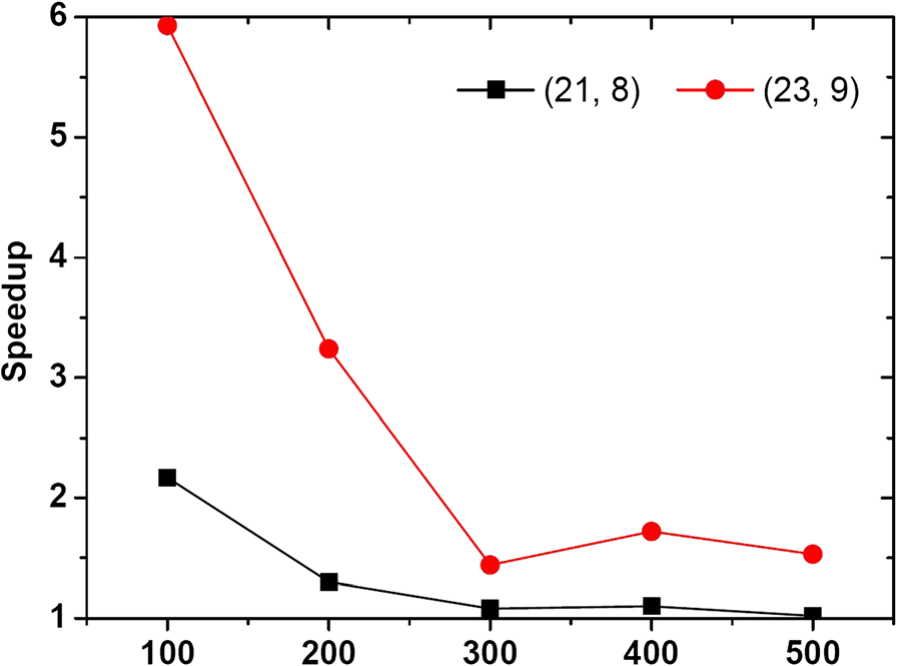
The effect of sequence length on the speedup for the DNA data. This figure shows the effect of sequence length on the speedup for the DNA data. We fix |Σ| = 4 and *q* = *t* = 20, and vary the sequence length *n* from 100 to 500. The tested algorithm is qPMS7 and PMS problem instances are (21, 8) and (23, 9)

Finally, we test the effect of the sequence length *n* on the speedup brought by RefSelect for existing algorithms. In the experiment, we fix |Σ| = 4 (DNA data) and *q* = *t* = 20, and vary *n* from 100 to 500; the tested algorithm is qPMS7 and PMS problem instances are (21, 8) and (23, 9). The results are shown in Fig. 7. Overall, the speedup increases with the decrease of *n*. This is because, according to (11) and Table 2, the smaller the value of *n*, the larger the difference between the number of candidate motifs for *E*_*i*_ < 1 and that for *E*_*i*_ ≥ 1.

The fact that RefSelect works better for short input sequences makes sense to motif discovery in next-generation or high-throughput sequencing data sets, such as ChIP-chip [29] and ChIP-seq [30] data sets. These data sets have a better resolution for containing motifs than the traditional promoter sequences. For example, the length of each sequence in ChIP-seq data sets is usually 200 base pairs, while the length of a promoter sequence is about 1000 base pairs. Therefore, RefSelect can bring a better practical time improvement of the pattern-driven PMS algorithms on the ChIP-seq data sets than that on the traditional promoter sequences.

### Assessment of RefSelect on large data sets

All experiments involved in the previous section focus on the data sets of small scale, namely the number of input sequences *t* is small. In recent years, with the rapid development of high-throughput technologies, which allows genome-wide identification of motifs, the data sets such as ChIP-seq [30] contain hundreds or more sequences. Thus, it is necessary to further assess the time performance and validity of RefSelect on large data sets.

First, we make the following settings in the experiment: set the maximum value of *t* as 600, as the ChIP-tailored version of MEME can effectively identify motifs by using 600 sequences randomly selected from the whole ChIP-seq data sets [31]; set *k* as 5 % × *t*, as most of the sequences in ChIP-seq data sets contain motif instances; set the sequence length *n* as 200, as the resolution that ChIP-seq sequences contain motifs is higher than that for traditional promoter sequences [9].

Since there is not an exact algorithm that can efficiently deal with large data sets, we assess the validity of RefSelect as follows. Let *N*_*original*_ denote the number of candidate motifs generated from the first *k* sequences in the original input sequences, and *N*_*improved*_ denote the number of candidate motifs generated from the *k* reference sequences selected by RefSelect. Then, we compute *N*_*original*_/*N*_*improved*_. A larger *N*_*original*_/*N*_*improved*_ indicates more candidate motifs can be reduced.

On the above basis, we get the running time of RefSelect and *N*_*original*_/*N*_*improved*_ on both the DNA and protein data sets, by varying *t* from 50 to 600. From the results shown in Table 6, we can find that: (1) RefSelect can quickly select reference sequences from these data sets, and its running time is independent of the alphabet size; (2) the running time of RefSelect increases with the number of input sequences *t* and exceeds one minute when tackling the task of *t* = 600; (3) RefSelect can still reduce the generated candidate motifs, especially for the protein data (large alphabet). Besides the running time of the whole RefSelect algorithm, we also list the running time of the first step of RefSelect, which shows that the first step is the bottleneck of the whole RefSelect algorithm.

**Table 6:** Assessment of RefSelect on large data sets

*t*	*k*	DNA Sequences			Protein Sequences		
		*time*	*time* ^*a*^	*N*_*original*_ */N*_*improved*_	*time*	*time* ^*a*^	*N*_*original*_ */N*_*improved*_
50	3	0.5 s	0.4 s	2.37	0.4 s	0.4 s	15.79
100	5	2.0 s	1.9 s	2.56	1.8 s	1.7 s	15.41
200	10	8.2 s	8.0 s	1.85	7.3 s	7.1 s	14.20
300	15	18.6 s	18.1 s	2.43	16.2 s	15.9 s	18.31
400	20	33.5 s	32.5 s	2.52	28.7 s	27.9 s	18.42
500	25	52.4 s	50.6 s	2.78	44.9 s	43.5 s	16.64
600	30	75.8 s	73.5 s	2.56	66.8 s	64.8 s	15.98

s: seconds; *time* and *time* ^*a*^: the running time of RefSelect and that of the first step of RefSelect; *N*_*original*_ and *N*_*improved*_: the number of candidate motifs generated from the first *k* original input sequences and that for the *k* reference sequences selected by RefSelect.

Second, we test the parallel version of RefSelect. It is pretty common that a ChIP-seq data sets contains more than one thousand sequences, and parallel processing is a good choice in this case. In the experiment, we use the protein data sets with *k* = 5 % × *t* and *n* = 200. We give the running time in Table 7 by varying *t* from 200 to 1600 and the number of threads from 1 to 8. We can find that the acceleration of RefSelect through parallel processing is obvious, and the speedup is almost linearly proportional to the number of threads.

**Table 7:** Running time of RefSelect using parallel processing

*t*	Number of threads			
	1	2	4	8
200	7.3 s	4.4 s	2.4 s	1.5 s
400	28.7 s	17.1 s	9.3 s	4.9 s
600	66.8 s	37.7 s	20.5 s	10.6 s
800	127.9 s	67.3 s	38.0 s	21.1 s
1000	204.4 s	106.7 s	59.5 s	34.0 s
1200	295.1 s	154.4 s	84.3 s	49.7 s
1400	401.7 s	211.9 s	118.0 s	68.9 s
1600	524.0 s	277.1 s	160.1 s	92.3 s

### Applicability of RefSelect

For the proper use of RefSelect, we summarize the applicability of RefSelect as follows.

1. RefSelect can accelerate such pattern-driven PMS algorithms that use random or the first *k* ≥ 2 sequences in the input as reference sequences to generate candidate motifs. For the efficient and recently proposed PMS algorithms, RefSelect is applicable for qPMS7, TravStrR and PMS8, but not for qPMS9, which does not use fixed *k* reference sequences to obtain *h*-tuples.
2. RefSelect can deal with large data sets containing hundreds or even more sequences.
3. The speedup brought by RefSelect for PMS algorithms is affected by the alphabet size. The larger the alphabet size, the larger the speedup.
4. The speedup brought by RefSelect for PMS algorithms is also affected by the sequence length *n*, which increases with the decrease of *n*.
5. RefSelect works better on the challenging instances with large *l* and *d*. For the challenging instances with small *l* and *d*, however, it is not necessary to use RefSelect, for they can be quickly solved by existing PMS algorithms.

Moreover, it is necessary to declare the following two points. First, the instability of the time performance is not reported in the previous literatures [19, 20, 23]. This is because we find that in their experimental data, the implanted motif instances differ from the motif in exact *d* positions. In this case, the probability of containing pairs of *l*-mers with *E*_*i*_ < 1 in the reference sequences is small, and accordingly the number of generated candidate motifs is also small. But it should be pointed out that, in reality motif instances differ from the motif in at most *d* positions, which leads to the execution time instability for some of the existing algorithms.

Second, although RefSelect is not applicable for qPMS9, which can solve challenging instances with larger *l* and *d* than previous algorithms, our research is still valuable. The reason is that qPMS9 cannot be used as a substitute for other PMS algorithms; we found in the experiments that qPMS9 sometimes exits unexpectedly with an out of memory error. Particularly, this phenomenon becomes frequent in dealing with challenging PMS instances of large (*l*, *d*) such as (*l*, *d*) = (21, 10) and (23, 11).

## Conclusions

We build the reference sequence selection problem and propose a method named RefSelect to select reference sequences for the pattern-driven PMS algorithms, in order to solve the problem that many pattern-driven PMS algorithms present execution time instability. RefSelect requires a small amount of storage space and is capable of selecting reference sequences efficiently and effectively. Also, the parallel version of RefSelect is provided for handling large data sets. For the state-of-the-art algorithms qPMS7, TravStrR and PMS8, RefSelect enables them steadily solve PMS problems in an efficient way without doing any modification to these algorithms.

Our work in this paper only focuses on selecting reference sequences for the pattern-driven PMS algorithms. It is recommended that further research be undertaken in selecting reference sequences for the iterative optimization algorithms of finding motifs in large data sets. These algorithms, such as MEME-ChIP [31], usually randomly select hundreds of sequences from a large input to make motif discovery, with a low chance of discovering infrequent motifs [32]. Thus, elaborate selection of sequences may help them obtain more motif information.
